# Virtual Reality Cardiac Surgical Planning Software (CorFix) for Designing Patient-Specific Vascular Grafts: Development and Pilot Usability Study

**DOI:** 10.2196/35488

**Published:** 2022-06-17

**Authors:** Byeol Kim, Phong Nguyen, Yue-Hin Loke, Vincent Cleveland, Xiaolong Liu, Paige Mass, Narutoshi Hibino, Laura Olivieri, Axel Krieger

**Affiliations:** 1 Department of Mechanical Engineering Johns Hopkins University Baltimore, MD United States; 2 Department of Computer Science University of Maryland College Park, MD United States; 3 Division of Cardiology Children's National Hospital Washington, DC United States; 4 Department of Surgery University of Chicago Chicago, IL United States

**Keywords:** virtual reality, congenital heart disease, surgical planning, usability study, heart, surgery

## Abstract

**Background:**

Patients with single ventricle heart defects receive 3 stages of operations culminating in the Fontan procedure. During the Fontan procedure, a vascular graft is sutured between the inferior vena cava and pulmonary artery to divert deoxygenated blood flow to the lungs via passive flow. Customizing the graft configuration can maximize the long-term benefits. However, planning patient-specific procedures has several challenges, including the ability for physicians to customize grafts and evaluate their hemodynamic performance.

**Objective:**

The aim of this study was to develop a virtual reality (VR) Fontan graft modeling and evaluation software for physicians. A user study was performed to achieve 2 additional goals: (1) to evaluate the software when used by medical doctors and engineers, and (2) to explore the impact of viewing hemodynamic simulation results in numerical and graphical formats.

**Methods:**

A total of 5 medical professionals including 4 physicians (1 fourth-year resident, 1 third-year cardiac fellow, 1 pediatric intensivist, and 1 pediatric cardiac surgeon) and 1 biomedical engineer voluntarily participated in the study. The study was pre-scripted to minimize the variability of the interactions between the experimenter and the participants. All participants were trained to use the VR gear and our software, CorFix. Each participant designed 1 bifurcated and 1 tube-shaped Fontan graft for a single patient. A hemodynamic performance evaluation was then completed, allowing the participants to further modify their tube-shaped design. The design time and hemodynamic performance for each graft design were recorded. At the end of the study, all participants were provided surveys to evaluate the usability and learnability of the software and rate the intensity of VR sickness.

**Results:**

The average times for creating 1 bifurcated and 1 tube-shaped graft after a single 10-minute training session were 13.40 and 5.49 minutes, respectively, with 3 out 5 bifurcated and 1 out of 5 tube-shaped graft designs being in the benchmark range of hepatic flow distribution. Reviewing hemodynamic performance results and modifying the tube-shaped design took an average time of 2.92 minutes. Participants who modified their tube-shaped graft designs were able to improve the nonphysiologic wall shear stress (WSS) percentage by 7.02%. All tube-shaped graft designs improved the WSS percentage compared to the native surgical case of the patient. None of the designs met the benchmark indexed power loss.

**Conclusions:**

VR graft design software can quickly be taught to physicians with no engineering background or VR experience. Improving the CorFix system could improve performance of the users in customizing and optimizing grafts for patients. With graphical visualization, physicians were able to improve WSS percentage of a tube-shaped graft, lowering the chance of thrombosis. Bifurcated graft designs showed potential strength in better flow split to the lungs, reducing the risk for pulmonary arteriovenous malformations.

## Introduction

Congenital heart disease is the most common birth defect found in nearly 1% of births worldwide [1]. Those patients who are diagnosed with single ventricle heart defect (SVHD), a rare type of congenital heart disease, experience mixed circulation of oxygenated and deoxygenated blood flows. Patients with SVHD receive 3 stages of life-saving surgery—Norwood, Glenn, and Fontan—to direct the deoxygenated blood flow to the lungs without going through the heart. Stage I, or the Norwood procedure, reconstructs the aortic arch, connecting it to the right ventricle, and a systemic-to-pulmonary artery shunt is placed [2]. At stage II, the Glenn procedure, a superior cavopulmonary anastomosis is created by connecting the superior vena cava (SVC) to the right pulmonary artery (PA) [3,4]. Stage III, the Fontan procedure, involves suturing a vascular graft between the inferior vena cava (IVC) to the PA to allow passive flow of venous blood to the lungs for oxygenation. When post-Fontan surgery circulation does not provide ideal hemodynamics, patients may have increased risk of elevated PA pressure, anatomic abnormalities of the PAs, atrial-ventricular valve regurgitation, and poor ventricular function [4].

Advances in medical imaging scanning and 3D-printing techniques have been showing great potential for customizing Fontan grafts. One of the customization approaches is known as tissue-engineered vascular grafts (TEVGs), which uses biocompatible material to facilitate the growth of neotissue, including collagen, vascular muscle, and endothelial cells [5,6]. One of the prominent strengths of the growth of neotissue is the patency [7], allowing an implanted graft to grow over time along with patients [8]. It is also believed to be more thrombo-resistant and less infectious than are comparable synthetic grafts [9]. These characteristics could support long-term benefits for Fontan procedures. TEVGs involve 3D-printing techniques, such as casting, electrospinning, and modular construction, that can fabricate any shape of a TEVG scaffold [10]. Since synthetic grafts are conventionally limited to specific designs (ie, cylindrical tube-shaped and bifurcated), being able to fabricate a scaffold allows for more patient-specific operations.

3D-printed scaffolds can be modeled using various approaches including computer-aided design (CAD) software [11], graft modeling software (such as SURGEM [12]), and unconstrained clay modeling [13]. CAD software is the most widely used tool for parameterizing a graft design [11,14,15]. Despite its popularity, CAD’s complex parametric design process requires extensive training and practice, which can be a significant challenge for physicians. SURGEM and unconstrained clay modeling are 2 great alternatives which enable physicians to perform the modeling tasks more easily and quickly. SURGEM is a tablet-based heart-specific surgical planning software [12]. It provides hole filling, stenosis repair, and Fontan graft design features. In SURGEM, the diameter, center line, and anastomosis region are defined, supporting the design of a cylindrical Fontan graft. The designed grafts using SURGEM may not match the size of the native IVC because they are limited to cylindrical designs [16]. Furthermore, since anatomies are complex and volumetric, lack of depth perception may challenge the design process. Unconstrained clay modeling involves molding physical clay onto a 3D-printed model of the total cavopulmonary connection (TCPC) anatomy [13]. This method does not require significant training to operate. However, relying on 3D-printed TCPC anatomy and clay makes it difficult for precise control, and small form changes can have dramatic consequences. Additionally, detailed viewing and reporting, design saving, and future edits are not straightforward with these techniques.

The ability to produce graft designs alone is not sufficient to optimize Fontan procedures. Without accounting for the flow inside each graft design, a patient may experience increased risk of medical complications. Multiple studies have emphasized the importance of a low indexed power loss (iPL) [17,18], a balanced hepatic flow distribution (HFD) [19,20], and a low nonphysiologic wall shear stress percentage (%WSS) [13]. High iPL is correlated with a greater chance of exercise intolerance [21], an unbalanced HFD is associated with pulmonary arteriovenous malformations [22], and low %WSS regions are associated with a higher chance of clot formation [23]. Evaluating these hemodynamic performances can be done using physical models or computational fluid dynamic (CFD) simulations. The physical setup entails 3D printing a modeled graft and running blood-mimicking fluid through it. Advanced imaging techniques, such as 4D flow magnetic resonance imaging, and optical imaging methods, such as particle image velocimetry [24], are used to measure the flow velocity field for computing WSS and HFD. iPL can be measured by pressure sensors at the boundaries on the printed grafts [25,26]. These approaches, however, require each design modification to be printed and tested. Thus, the physical setup for measuring hemodynamic performances is labor and time intensive. Physical testing is also limited by spatial resolution, signal noise, and segmentation errors. As a mathematical method for calculating fluid flow, CFD can reduce or even overcome these limitations [27,28]. It can visualize multiple flow properties inside any shape of grafts on a computer without the need to purchase any devices or print the actual models. The accuracy of CFD simulations is widely recognized and has been validated by multiple in vivo and in vitro studies [25,29-31]. However, there needs to be further development in tools that bridge 3D modeling and CFD. Most available tools for performing these tasks are complicated and require hours of training. In our previous study, we developed our first prototype of virtual reality (VR) vascular graft design software, CorFix [32]. The first prototype of CorFix integrated diagnosis, tube-shaped graft design, free-form graft design, and 3D export features. The diagnosis feature consisted of rotation, zoom in and out, anatomy clipping, annotation, and screenshot. The free-form graft design included pushing and pulling methods for manipulating a surface mesh of a designed tube-shaped graft. Even when the software was evaluated by engineers with extensive CAD training, CorFix outperformed CAD software in time and graft design quality. In this study, we developed a significantly improved second version of CorFix, modifying the VR interface and adding bifurcated graft design, design export and import, and CFD visualization features. Although engineers were proven capable of completing the graft design task, we focused here on enabling and evaluating the ability for physicians to manage the design task. By evolving the CorFix software, we expect to remove the uncertainty around the evaluation of surgical feasibility and preference. We also anticipate reducing communication and discussion times for patient-specific surgical planning by avoiding the back-and-forth communication between multiple parties. There are 2 objectives to this study: (1) to evaluate the use of the software by medical doctors and engineers and (2) to explore the impact of viewing hemodynamic simulation results in numerical and graphical formats. Our study included usability testing and design performance evaluations where we compared CorFix designs created by 4 medical doctors and 1 biomedical engineer for an actual surgical case.

## Methods

### Ethics Approval

This study was approved by the investigational review board at Children’s National Hospital (reference number: Pro00009721).

### Medical Image Selection and Acquisition

**Figure 1:**
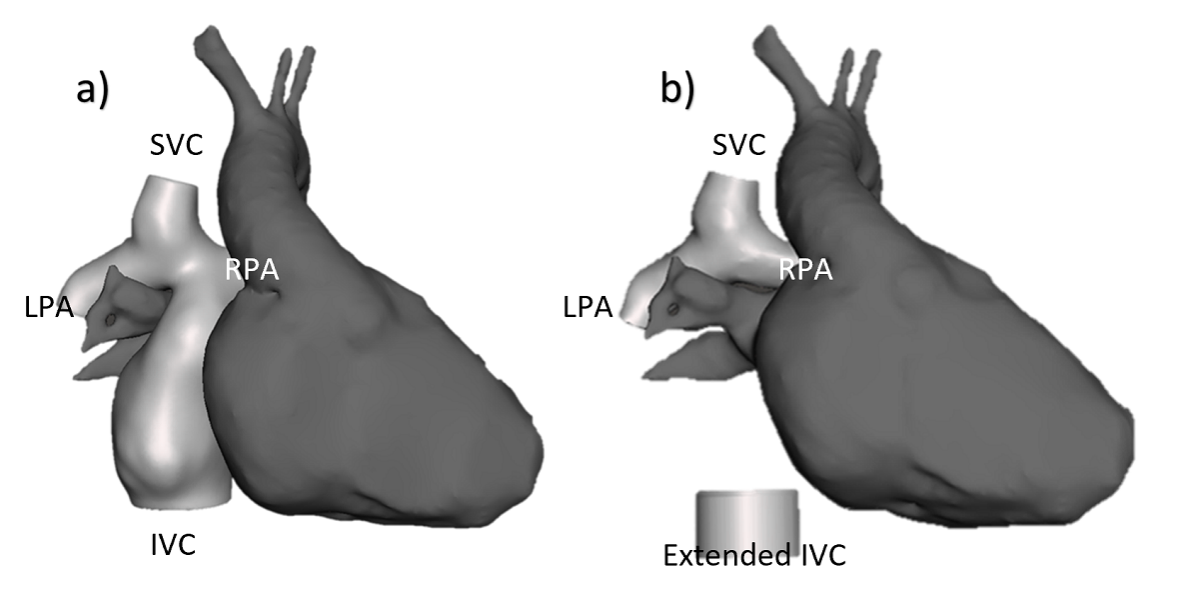
Patient’s Fontan anatomy. (a) The 3D models of the anonymized patient anatomy: heart (dark gray) and total cavopulmonary connection (light gray). (b) Patient anatomy with Fontan IVC to Glenn conduit removed and 10-mm inferior extrusion on the IVC. LPA: left pulmonary artery; IVC: inferior vena cava. RPA: right pulmonary artery; SVC: superior vena cava.

One anonymized post-Fontan procedure imaging data set was acquired via magnetic resonance imaging. The data set was exported as a DICOM file and then manually segmented into two 3D models using Mimics software (Materialise): a (1) TCPC model and (2) a heart model without the TCPC anatomy (Figure 1a). CFD simulation was performed on the TCPC anatomy to evaluate its hemodynamic performance. For the experiment, the sutured vascular graft was virtually removed from the TCPC anatomy, resulting in 2 separate anatomies including the IVC and Glenn (ie, PA and SVC). The native IVC surface was extruded 10 mm inferiorly using SolidWorks software (Dassault Systèmes) to show the direction of the native IVC (Figure 1b).

### CorFix Development

The VR surgical planning software, CorFix, was developed based on the Unity 3D engine. The software-running platform was an Alienware Aurora R8 (Dell) with an Intel Core i7-9700 processor, a NVIDIA GeForce RTX-2080Ti, and 16 GB of RAM. An Oculus Rift S was used for displaying CorFix in full-immersive VR. Touch controllers (Oculus Rift) were integrated into the system for interacting with the interface. CorFix was previously designed to perform simple diagnosis (ie, zoom, rotation, label, ruler, and clipping) and modeling (ie, cutting vessels, parametric modeling, and free-form modeling) tasks. This version of CorFix had a modified user interface to accommodate clinicians untrained in VR, modeling software (eg, CAD), or CFD. The interface was adapted to allow users to intuitively design patient-specific vascular grafts in a short amount of time and integrate image analysis in the workflow.

#### CorFix Interface for Graft Design

**Figure 2:**
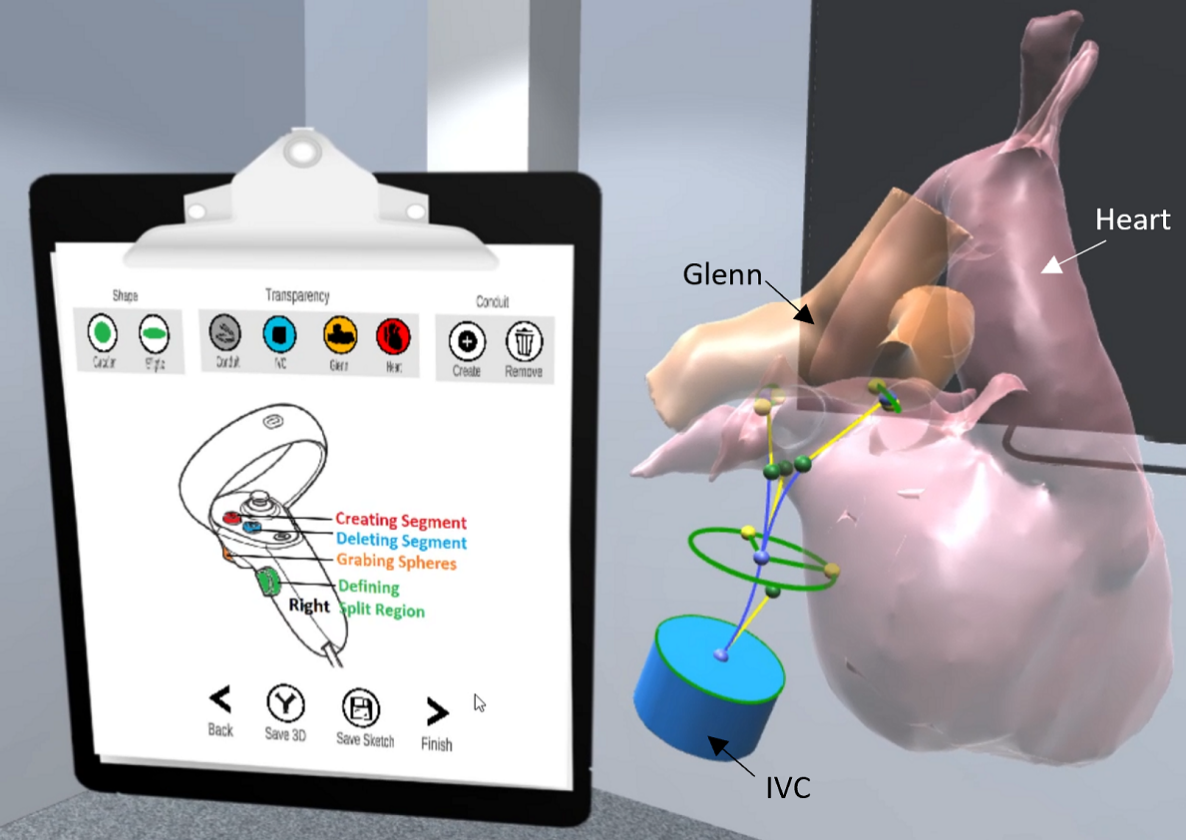
Screenshot of a user creating a bifurcated graft using CorFix. IVC: inferior vena cava

The Corfix interface was designed to support simple memory recall, allowing for a short, 1-time, 10-minute tutorial. A virtual clipboard was used as an access point for menu and Oculus controller information. The top row contained icons that support the designing of a tube-shaped or bifurcated graft. Icons were designed to match the color and shape of the corresponding geometry and anatomy. In the center of the clipboard, a diagram of the Oculus controller and its functionality were visualized. The bottom row contained menus that are necessary when the design process is completed (Figure 2). A “Save 3D” menu option was included for exporting the designed graft to a 3D-formatted OBJ file. The “Save Sketch” menu was included as a newly developed feature to save the current graft design for future edits.

#### Design Export and Import Feature

The import process saves all information needed to reconstruct the graft designs using the same algorithm used to construct the conduits. It first saves the transform information of the heart, Glenn, and the graft. The location and radii of the Bezier curve are then stored. These data are then exported into 1 CSV (comma-separated value) file in the aforementioned order. The design import feature works by parsing the saved file from top to bottom and then reconstructing the scene in that order.

#### Bifurcated Design Feature

**Figure 3:**
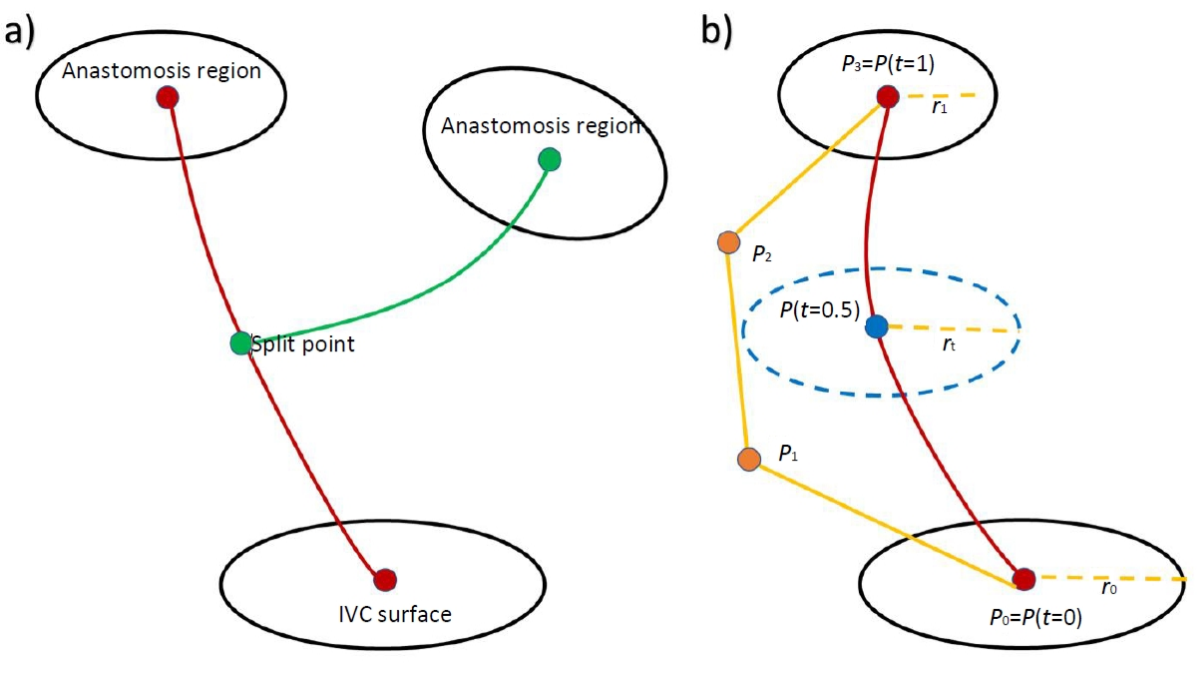
Schematic of Fontan graft designs. (a) Minimum design parameters for a bifurcated graft and (b) the cubic Bezier curve and radii interpolation diagram. IVC: inferior vena cava.

The minimum design parameters for a bifurcated graft were 2 anastomosis regions and 1 split region (Figure 3a). For defining the anastomosis location, a center blue sphere was modified. Two yellow spheres were located near their respective geometry control points for defining the radii of the ellipse. These yellow spheres were grabbed and adjusted by the Touch controller. Subsequently, through use of a polar equation for an ellipse, multiple radii along the ellipse were then calculated and stored.

These calculated points were connected to the center of the ellipse to make triangular meshes, forming a surface. Two cubic Bezier curves were used to define the pathways and girths of the bifurcated graft. The first Bezier curve used the center of the native IVC surface and a user-defined anastomosis region. The second Bezier curve used the center of another anastomosis region and a user-specified split region of the graft. The formula for the pathways was as follows:

P(*t*) = *P*_0_ (1 + *t*)^3^+*P*_1_ (3*t*(1 – *t*)^2^)+*P*_2_ (3*t*^2^ (1 – *t*)) + *P*_3_ (*t*^3^) **(1)**

where *P*_0_ and *P*_3_ are anchor points which represent the center points of 2 different surfaces; and *P*_1_ and P_2_ are handles which define the direction and strength of the pathways, with the variable *t* ranging from 0 to 1 (Figure 3). Users were given an option to add as many anchor points as they wanted for more precise and complex control of the pathways. Adding an additional anchor point splits a single Bezier curve into 2 cubic Bezier curves. Two adjacent handles were automatically created at each anchor point. Connecting elliptic meshes and the native IVC surface along the pathway required the 3 following steps of interpolation:

∆ = *r*_1_ – *r*_0_
**(2)**

*f*(*t*)
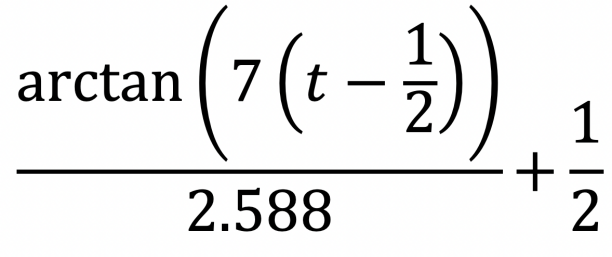
**(3)**

*r_t_* = *r*_0_ + ∆ ∙*f*(*t*) **(4)**

where ∆ is the difference between the radii from one center point to another. For example, *r*_1_ could be one of the radii from the anastomosis region and *r*_0_ could be one of the radii from the native IVC surface. *f*(*t*) is the interpolation adjustment factor at *t*. The letter *t* represents any location on the Bezier curve. The center points of *r*_0_ and *r*_1_ are defined as 0 and 1 for *t*.

#### Hemodynamic Simulation Visualization

**Figure 4:**
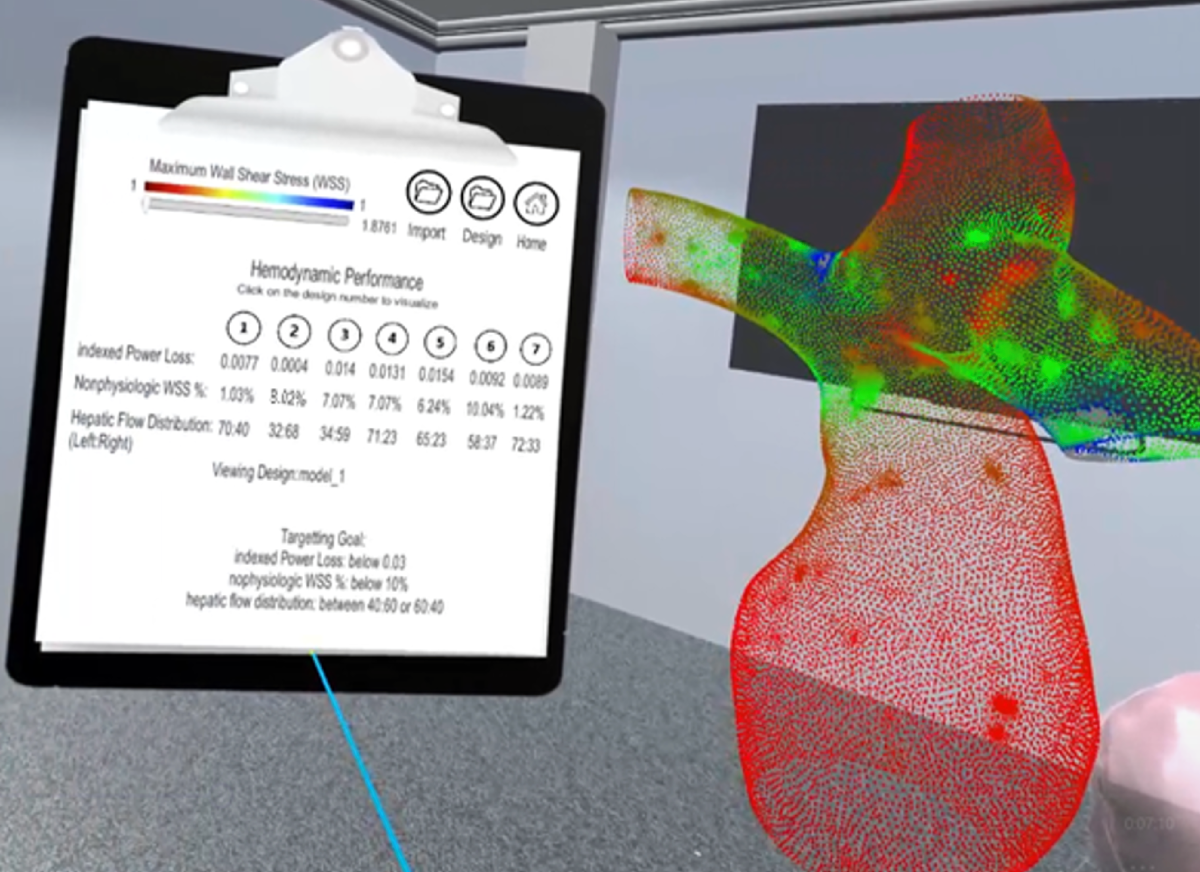
Screenshot of the hemodynamic simulation results of clipboard and point cloud rendering using the percentage of nonphysiologic wall shear stress output values.

Hemodynamic simulation results were outputted in .h5 format. A data import and transform script was developed using MATLAB (MathWorks) since the .h5 format is not supported in Unity. The script consisted of 3 parts: data size, hemodynamic performance summary, and raw WSS values. The data size rows summarized the number of graft designs that were simulated and the total length of the raw WSS values. The hemodynamic performance summary contained information on iPL, %WSS, and HFD on each graft. The raw WSS values are composed of actual WSS values on each x, y, and z coordinate of a graft. These parts are concatenated into 1 CSV file, which is then imported into Unity. As a default, minimum and maximum WSS are set to 0 and 1 
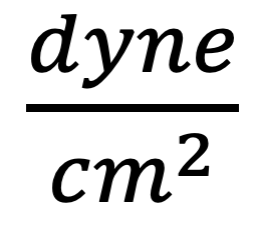
, respectively. The maximum WSS can be changed by scrolling a slider on the clipboard towards the right. The maximum threshold of the slider is automatically identified by calculating the biggest WSS value from the CSV file. All points with nonzero WSS values are rendered using graphics processing unit acceleration and display relevant data regarding that point cloud. The graphics processing unit acceleration approach enables real-time point cloud rendering that corresponds to the slider. The rendered point cloud was grabbable and rotatable for users to study in detail or to match its orientation to their view of current designed graft (Figure 4).

### CFD Simulations

#### Benchmark Hemodynamic Performance Parameters

The hemodynamic performance parameters included iPL, %WSS, and HFD. iPL is a dimensionless value of a pressure difference between the Fontan graft and the PA. It is normalized using a patient’s body surface area. High iPL values have an increased chance of deteriorated cardiac performance and exercise capacity [33]. The iPL is calculated as follows:

iPL = 
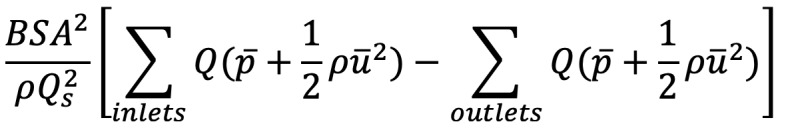

**(5)**

where BSA is the body surface area of the patient, 
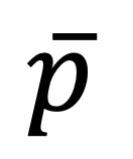
 is the static pressure, *ρ* is the density of the blood, 
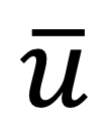
 is the velocity, *Q* is the flow rate, and *Q_s_* is the systemic venous flow that is equivalent to the sum of all inlet flow rates. The WSS is defined as a force created against the surface of the graft by the blood. A healthy physiologic range of venous WSS falls between 1 and 10 *dyne*/cm^2^. If WSS is below the lower threshold, there could be an increased chance in thrombus formation on the surface of the graft [34]. The ratio of the areas that are below 1 *dyne*/cm^2^ has been identified as the nonphysiologic regions. Its percentage against the total area was calculated for %WSS as follows:



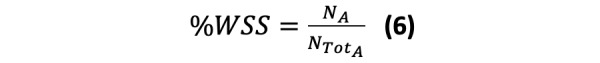



where *N_A_* is the number of WSS values below 1 *dyne*/cm^2^ on the graft, and 
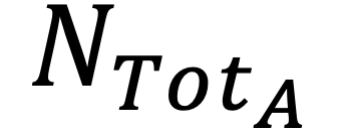
 is the total number of WSS values. The HFD is a ratio of the flow split to the PA from the Fontan. Unbalanced flow split may result in higher risk of pulmonary arteriovenous malformation [35]. HFD was calculated using a 1-way coupling Lagrangian particle-tracking method. This involves releasing massless infinitesimal particles at the IVC (N_IVC_). The number of particles that pass through each side of the PA (N_LPA_ and N_RPA_) is then counted as follows:



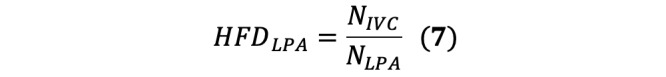





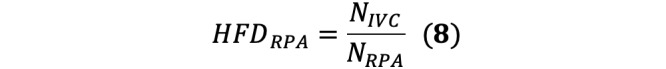



The number of total particles varies and depends on the surface area of the inlets. The particles are equally spaced from each other. This study set the healthy ranges of each benchmark parameter as below 0.03 for iPL, below 10% for %WSS, and within the range of 40% to 60% for the HFD ratio.

#### CFD Simulations

Ansys Fluent 19 (ANSYS Inc) was used to make extensions at inlet and outlet boundaries. The inlet, IVC, and SVC, were extruded by 10 times their largest diameter. The outlets, that is the left and right PA, were extruded by 50 mm. These extensions acted as a mechanism for developing a stable blood velocity profile. The CFD simulation was performed by solving steady 3D Navier-Stokes equations with Newtonian fluid and rigid wall assumptions. A calculation for the Reynolds number was implemented to assess the laminar flow of a patient’s anatomy.

### Pilot Usability Testing

#### Recruitment

The institutional review board at the Children’s National Hospital in Washington, DC, approved this study. The study was advertised by sending emails to the groups of residents, fellows, cardiac specialists, and medical engineers. A total of 5 voluntary participants were recruited including 1 fourth-year resident, 1 third-year cardiac fellow, 1 pediatric intensivist, 1 pediatric cardiac surgeon, and 1 biomedical engineer. All participants gave informed consent prior to their participation.

#### Experimental Process

Before the experiment, all participants were queried about their knowledge on the Fontan procedure and vascular grafts. Those who did not have a strong understanding about the topics were given a short tutorial. The tutorial covered anatomy of patients with SVHD, surgical repair for SVHD, and the shapes of Fontan grafts in 3 PowerPoint (Microsoft) slides. All participants then received a tutorial on the 3 benchmark parameters that would be calculated to identify the performance of their Fontan graft designs. This tutorial did not include any information about the relationships between each benchmark parameter and the graft design parameters. The participants were informed that %WSS is negatively correlated with iPL. Healthy ranges of each benchmark parameter were visually provided inside the VR environment as a reference. The last tutorial was for the CorFix interface and took about 10 minutes. None of the participants had prior experience with VR, requiring the CorFix tutorial to include information about the hardware (Oculus). During the CorFix tutorial, participants wore the gear and went through the following topics with verbal feedback: importing anatomies, interacting with the anatomies, designing basic tube-shaped and bifurcated Fontan grafts, making anatomies transparent, visualizing CFD results, and modifying the existing tube-shaped design.

**Figure 5:**
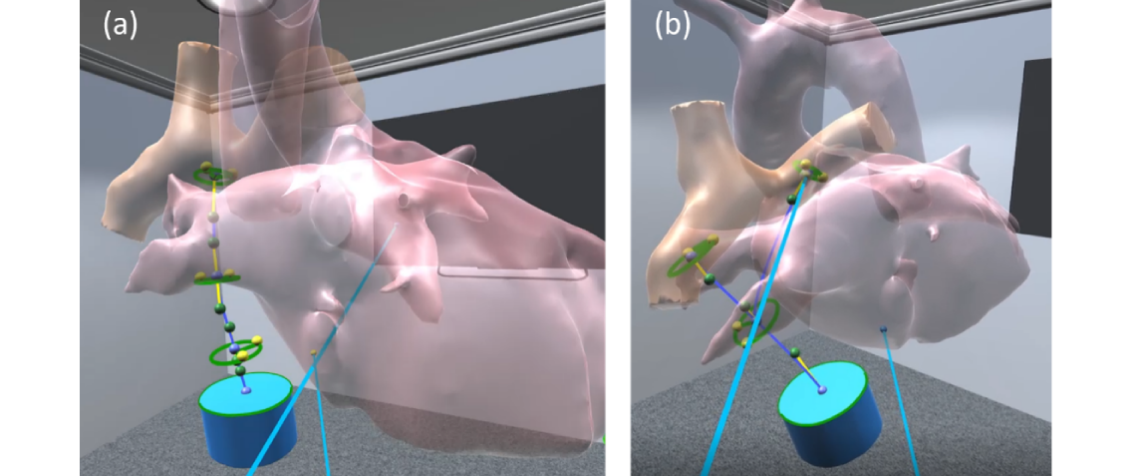
A participant creating a (a) tube-shaped and a (b) bifurcated Fontan graft on CorFix during the experiment.

After it was confirmed there were no further questions about the VR gear or CorFix software, participants created and exported 3D models of 1 bifurcated and 1 tube-shaped Fontan graft (Figure 5). There was no time limit for designing the graft. The second part of the experiment involved evaluating hemodynamic performances of the patient’s anatomy along with 6 other anatomies that had 1 design parameter variation. The variations included the suturing region angled leftward, rightward, and upward; having a smaller anastomosis region; and offsetting the suturing region toward the left and right. None of these anatomies were optimal in any of the 3 benchmark parameters. All participants made individual decisions about the designs to find patterns or improvements for further modifying a previously designed tube-shaped graft. The participants were not required to modify their design. Three hard-printed surveys were provided at the end of the design modification. The entire experiment was scripted to provide a uniform experience.

#### Surveys

All participants filled out a digital demographic survey prior to the experiment, including questions about their position, level of VR experience, knowledge and experience on the Fontan procedure, and the level of training on fluid dynamics. Three hard-printed surveys were provided at the end of the study. The System Usability Scale (SUS) and the Usefulness, Satisfaction, and Ease of Use Questionnaire (USE) were used to measure the usability of the system. To identify the level of sickness when using VR gear, the Simulator Sickness Questionnaire (SSQ) was provided.

## Results

### Design Times

**Figure 6:**
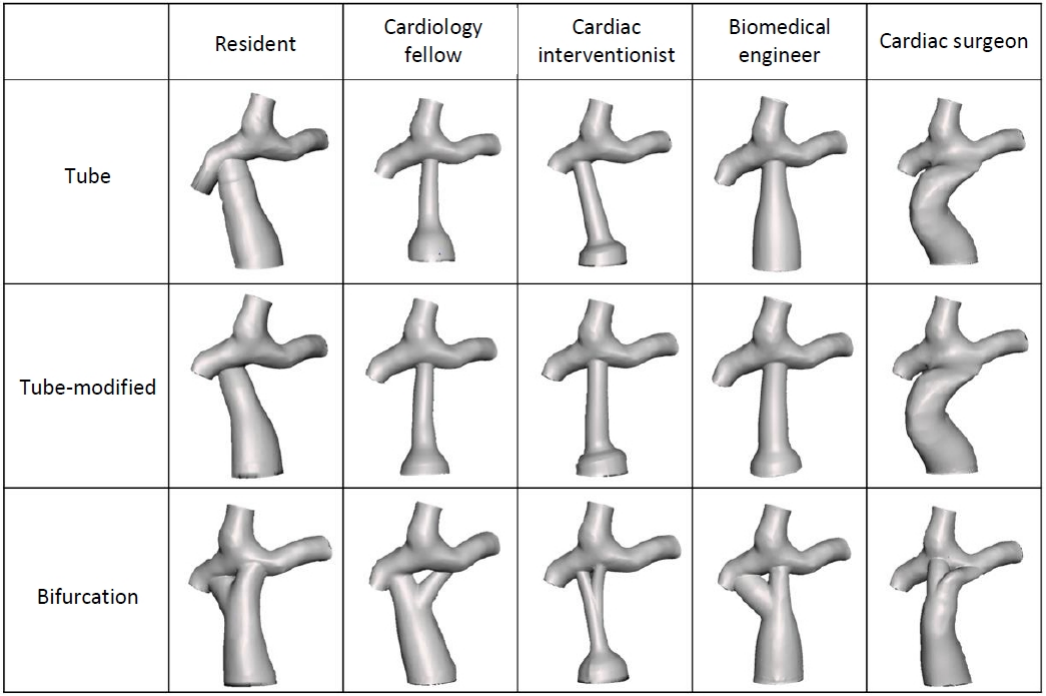
Summary figure of the Fontan graft designs.

Participants spent an average of 5.49 minutes creating 1 tube-shaped graft and 13.40 minutes creating 1 bifurcated graft. An average of 2.92 minutes was spent modifying the tube shape after it was created. This time includes reviewing the native patient model and the 6 design variations. The summary of design times and actual designs are provided in Table 1 and Figure 6.

**Table 1:** Summary of graft design and modification times.

	Tube-shaped	Modified tube-shaped	Bifurcation
Time (min), mean (SD)	5.49 (2.35)	2.92 (1.67)	13.40 (3.48)
Minimum time (min)	2.50	2.49	9.45
Maximum time (min)	8.10	5.07	16.57

### Hemodynamic Performance

#### Native Fontan Patient

**Figure 7:**
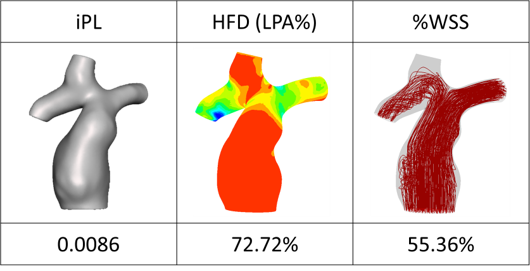
Hemodynamic performance of the provided Fontan data set without any modifications. %WSS: percentage of nonphysiologic wall shear stress; HFD: hepatic flow distribution; iPL: indexed power loss; LPA: left pulmonary artery.

The patient Fontan data set without modifications showed suboptimal hemodynamic performance. with 55.36% of the Fontan anatomy under nonphysiologically optimal WSS, unbalanced HFD with 72.72% of hepatic flow going to the left PA, and an iPL of 0.0086, indicating minimal flow change within the anatomy (Figure 7).

#### Tube-Shaped and Bifurcated Grafts

**Figure 8:**
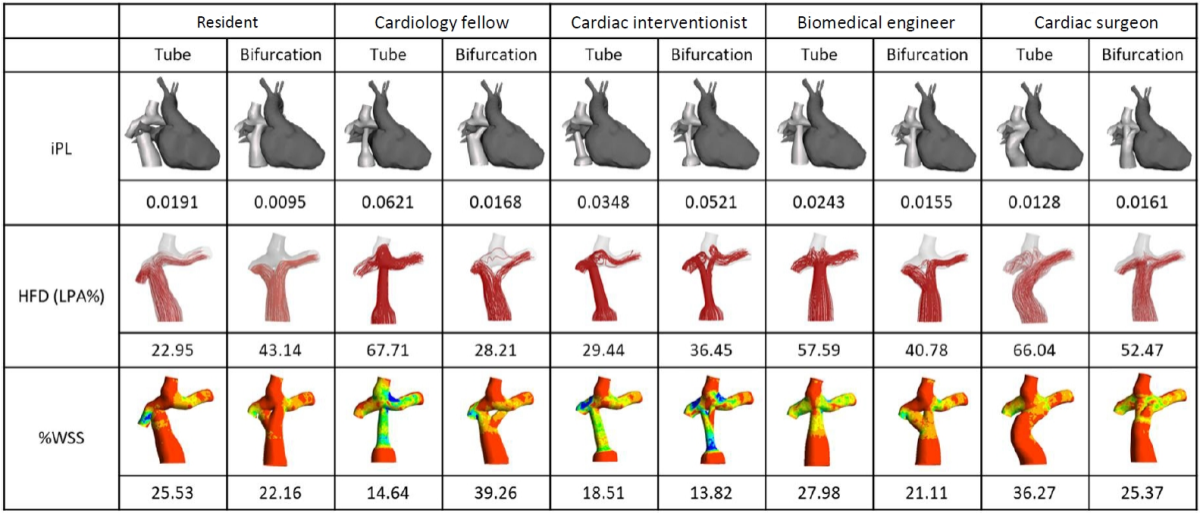
Summary of computational fluid dynamics simulations on the participants’ Fontan graft designs. %WSS: percentage of nonphysiologic wall shear stress; HFD: hepatic flow distribution; iPL: indexed power loss; LPA: left pulmonary artery.

Each participant produced 1 tube-shaped and 1 bifurcated Fontan graft. CFD simulations were performed on each of the graft designs. The detailed hemodynamic results are provided in Figure 8. Regardless of the shape of the graft, all participants were able to create designs that were much lower in %WSS compared to that of the surgical case. However, none of the designs were under the safe range of 10% or below. The bifurcated Fontan graft generally showed an optimal range of HFD, between 40% and 60%. All graft designs had higher iPL values than did the native Fontan surgical case.

#### Tube-Shaped Graft Modification

**Figure 9:**
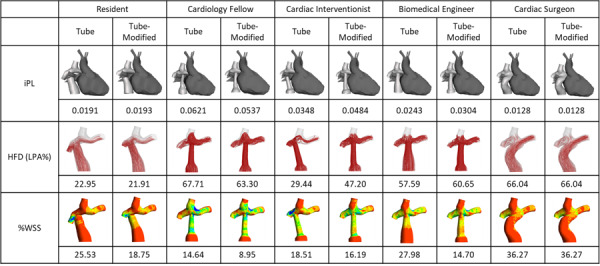
Summary table of computational fluid dynamic values after participants were presented with a set of prompt design variations; the %WSS values improved. %WSS: percentage of nonphysiologic wall shear stress; HFD: hepatic flow distribution; iPL: indexed power loss; LPA: left pulmonary artery.

All participants were asked to review 7 Fontan graft design variations based on the native Fontan surgical case. None of 7 design variations were considered optimal for the patient. The variations were created to assist the participants in identifying important design parameters that contribute to each hemodynamic benchmark parameter. After evaluating the design variations, the participants were given the freedom to modify their tube-shaped graft design to attempt to optimize the hemodynamic parameters. All those who modified their tube-shaped Fontan graft were able to reduce %WSS with an average improvement of 7.02%, ranging from 2.32% (cardiac interventionalist) to 13.28% (biomedical engineer; Figure 9).

### Surveys

CorFix scored an average of 57 on the SUS questionnaire with a minimum score of 42.5 and a maximum of 67.5. The average SUS value suggests that the usability of our prototype was marginal. The average total score of USE (Table 2) for all participants was 4.38 out of a maximum of 7. This indicates that CorFix provides a good degree of usefulness, satisfaction, and ease of use. The results for the 4 dimensions associated with USE were (1) usefulness (mean 3.75, SD 1.03), (2) ease of use (mean 4.47, SD 1.38), (3) ease of learning (mean 5.10, SD 1.13), and (4) satisfaction (mean 4.60, SD 1.64).

The SSQ (Table 3) showed that using VR for designing, reviewing, and modifying Fontan grafts in less than 30 minutes could still cause a high level of nausea, oculomotor, and disorientation problems: on average, participants gave 11.45, 24.26, and 16.70 for each parameter of SSQ, respectively. The SD was 10.45, 26.48, and 30.18, respectively. The high SD of disorientation is due to 3 out of 5 participants reporting no disorientation problems.

**Table 2:** Summary table for the Usefulness, Satisfaction, and Ease of Use Questionnaire.

	Usefulness	Ease of use	Ease of learning	Satisfaction	Overall
Score, mean (SD)	3.75 (1.03)	4.47 (1.38)	5.10 (1.13)	4.60 (1.64)	4.38 (1.10)
Maximum score	2.63	2.36	3.25	1.71	2.57
Minimum score	5.38	5.64	6.00	5.71	5.53

**Table 3:** Summary table for the Simulator Sickness Questionnaire survey.

	Nausea	Oculomotor	Disorientation
Score, mean (SD)	11.45 (10.45)	24.26 (26.48)	16.70 (30.18)
Maximum score	0	0	0
Minimum score	28.62	68.22	69.60

## Discussion

All participants were able to successfully design patient-specific conduits using the VR software with limited training. Although none of the participants had VR experience and CorFix was rated with marginal acceptable usability, designing tube-shaped and bifurcated grafts took less than 6 and 14 minutes, respectively. We used the time spent on a task as a surrogate for task difficulty and assessment of user adoption since there is sound literature indicating that among adult learners, time spent on a task is commensurate with task difficulty [36,37]. Even with design modification, less than 20 minutes could be spent to plan a Fontan procedure for each patient. Given the busy workload of surgeons and the urgent nature of patient care, being able to evaluate and customize a surgery for a patient in less than 20 minutes seems advantageous for the current surgical workflow. The study results mirror other recent studies for other surgical procedures demonstrating that VR is feasible and potentially useful but that satisfaction is limited by the technical limitations of the devices and the experience of disorientation [38-40].

All participants expressed that if a real-time hemodynamic analysis of their designs were available, they would be able to better pinpoint the flaws of their designs. We, therefore, plan on further developing the CorFix software to add real-time simulation and visualization features. Our system has implemented button and pointer color changes and tactile feedback (ie, vibration) to bolster the interactivity inside the virtual scene. However, many participants struggled with depth perception and interactivity. Grabbing design control points or even clicking buttons on the virtual menu were frequently observed. Developing a feature or a device that could better support tactile feedback may enhance the usability and the innate learnability of the software.

The bifurcated graft designs were more successful in improving the hepatic flow distribution to a healthy range compared to the tube-shaped graft designs. During the experiment, all participants were asked to review 7 different tube-shaped Fontan grafts, which were derived from the actual surgical case although none of these design variations were surgically optimal. We hypothesized that the participants would be able to find patterns between design parameters and hemodynamic performance. Our study showed that when participants decided to modify their designs after reviewing other cases, they were able to design a more optimal graft by lowering %WSS. On average, %WSS was reduced by 7.02%. A biomedical engineer with a strong fluid dynamics education background showed the maximum %WSS reduction of 13.28%. Considering how lower %WSS is related to a lower risk of thrombosis for Fontan grafts, this design could provide a significant long-term improvement for the patient. We therefore infer that showing problematic regions in color, like a contour map, may help doctors without an engineering background to sufficiently identify low %WSS. iPL and HFD improvements were not consistent throughout the participants. Unlike %WSS, these hemodynamic parameters were presented only in numerical format. We hypothesize that with supplementary graphical visualization, users may be able to improve iPL and HFD more easily.

With the development of graft modeling and evaluation software like CorFix, physicians may be able to easily customize Fontan grafts and find an optimal graft configuration for long-term benefits. We plan to further develop CorFix by adding real-time CFD simulation and automatic graft optimization features for bolstering the graft design and evaluation process. Our next study will incorporate many of these changes and focus on recruiting more cardiac surgeons and testing against a larger number of patient surgical cases.

This study had a small sample for recruitment due to the limited number of doctors and their time availability despite 3 months of advertising and 2 additional months during the data collection period. We were able to include individuals with various levels of medical experience, which provides a broad spectrum of users and supports important preliminary insights. Our future study will involve greater participation and a larger number of patient cases to supplement the current results.

This paper reports the design of a VR software for patient-specific designs of vascular grafts that demonstrated feasibility and initial usability in a pilot usability study. All participants were able to create patient-specific graft designs with minimal training, needing on average only 5.49 minutes to design 1 tube-shaped graft and 13.40 minutes to design 1 bifurcated graft. Participants rated the design software with a good degree of usefulness, satisfaction, and ease of use. Further design improvements are needed to visualize hemodynamics during the design process, and a larger study is required to fully compare the VR design to current state-of-the-art surgical procedures.

## Abbreviations

%WSS: percentage of nonphysiologic wall shear stress
CFD: computational fluid dynamics
CSV: comma-separated value
HFD: hepatic flow distribution
iPL: indexed power loss
IVC: inferior vena cava
SSQ: Simulator Sickness Questionnaire
SUS: System Usability Scale
SVC: superior vena cava
TCPC: total cavopulmonary connection
USE: Usefulness, Satisfaction, and Ease of Use Questionnaire
VR: virtual reality
WSS: wall shear stress
